# A Newly Isolated Thermostable Lipase from *Bacillus* sp.

**DOI:** 10.3390/ijms12052917

**Published:** 2011-05-04

**Authors:** Fairolniza Mohd Shariff, Raja Noor Zaliha Raja Abd. Rahman, Mahiran Basri, Abu Bakar Salleh

**Affiliations:** 1 Enzyme and Microbial Technology Research, Faculty of Biotechnology and Biomolecular Sciences, University Putra Malaysia, 43400 UPM Serdang, Selangor, Malaysia; E-Mails: ferrol2506@gmail.com (F.M.S.); abubakar@biotech.upm.edu.my (A.B.S.); 2 Enzyme and Microbial Technology Research, Faculty of Science, University Putra Malaysia, 43400 UPM Serdang, Selangor, Malaysia; E-Mail: mahiran@science.upm.edu.my

**Keywords:** *Bacillus* sp. strain L2, thermostable lipase, cloning, sequencing, molecular expression, characterization

## Abstract

A thermophilic lipolytic bacterium identified as *Bacillus* sp. L2 via 16S rDNA was previously isolated from a hot spring in Perak, Malaysia. *Bacillus* sp. L2 was confirmed to be in Group 5 of bacterial classification, a phylogenically and phenotypically coherent group of thermophilic bacilli displaying very high similarity among their 16S rRNA sequences (98.5–99.2%). Polymerase chain reaction (PCR) cloning of L2 lipase gene was conducted by using five different primers. Sequence analysis of the L2 lipase gene revealed an open reading frame (ORF) of 1251 bp that codes for 417 amino acids. The signal peptides consist of 28 amino acids. The mature protein is made of 388 amino acid residues. Recombinant lipase was successfully overexpressed with a 178-fold increase in activity compared to crude native L2 lipase. The recombinant L2 lipase (43.2 kDa) was purified to homogeneity in a single chromatography step. The purified lipase was found to be reactive at a temperature range of 55–80 °C and at a pH of 6–10. The L2 lipase had a melting temperature (Tm) of 59.04 °C when analyzed by circular dichroism (CD) spectroscopy studies. The optimum activity was found to be at 70 °C and pH 9. Lipase L2 was strongly inhibited by ethylenediaminetetraacetic acid (EDTA) (100%), whereas phenylmethylsulfonyl fluoride (PMSF), pepstatin-A, 2-mercaptoethanol and dithiothreitol (DTT) inhibited the enzyme by over 40%. The CD spectra of secondary structure analysis showed that the L2 lipase structure contained 38.6% α-helices, 2.2% ß-strands, 23.6% turns and 35.6% random conformations.

## 1. Introduction

Lipolytic enzymes are currently attracting significant attention because of their biotechnological potential. Most of the lipases used in industry are microbial enzymes, of both fungal and bacterial origin [1]. Lipases are an important group of enzymes both physiologically and commercially as the use of lipases for a variety of biotechnological applications is rapidly and steadily increasing [2]. In general, lipases have promising applications in organic chemical processing, detergent formulation, synthesis of biosurfactants, the agrochemical industry, paper manufacture, nutrition, cosmetics and pharmaceutical processing [3].

Thermostability is a desirable characteristic because it may allow the enzyme to endure conditions in industrial processes that use relatively high temperatures and/or organic solvents. Furthermore, enzymes from thermophilic bacteria have been found to be generally more resistant to denaturation than their mesophilic counterparts [4]. In this paper, identification of an industrially important lipase producer was conducted to determine its phylogenetic position in systematic microbiology. We also described a method for the rapid cloning of the lipase gene from thermophilic *Bacillus* sp. L2 using a set of consensus primers and Polymerase chain reaction (PCR) techniques, followed by expression and purification of the recombinant L2 lipase gene, and some properties of the purified enzyme including the circular dichroism (CD) spectroscopy studies.

## 2. Results and Discussion

### 2.1. Bacterial Identification

**Figure 1.**
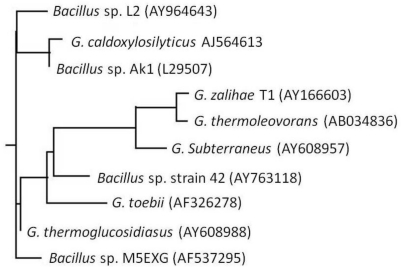
Rooted phylogenetic tree showing the relationship of isolate *Bacillus* sp. L2 to other *Bacillus* and *Geobacillus* species with their accession numbers.

*Bacillus* sp. L2 is an aerobic, non-motile Gram positive rod able to grow at a temperature range between 40–80 °C with an optimum growth at 70 °C and optimum pH of 7. The 16S rDNA nucleotide sequence of this strain has been deposited into GenBank data library and assigned the accession number AY 964643. BLAST results showed that *Bacillus* sp. L2 has highest homology with *G. thermoglucosidiasus* (AY 608988) with a significant value of 99% homology. From the phylogenetic tree and the G + C contents of 16S rRNA sequences, *Bacillus* sp. L2 was confirmed to be in Group 5 of bacterial classification (Figure 1). Group 5 is a phylogenically and phenotypically coherent group of thermophilic bacilli displaying very high similarity among their 16S rRNA sequences (98.5–99.2%) [5]. *Bacillus* sp. L2 was found to cluster closely with two other strains, *G. caldoxylosilyticus* (AJ 564613) and *Bacillus* sp. Ak1 (L 29507). The results from BLAST by NCBI also showed that *Bacillus* sp. L2 has 98% homology with these two strains, and also 99% homology with *G. thermoglucosidiasus* (AY 608988). However, from the phylogenetic tree, it was found that this *Bacillus* sp. L2 did not cluster closely with *G. thermoglucosidiasus* (AY 608988). So far, there are no reports on lipase production from either the *G. thermoglucosidiasus* or the *G. caldoxylosilyticus* species.

### 2.2. Isolation and PCR Cloning of the Thermostable L2 Lipase Gene

A fragment of lipase gene 300 bp in size was obtained using a pair of highly degenerate primers (Lip F2 and Lip R2). This lipase gene fragment showed the highest homology with the *B. stearothermophilus* P1 lipase gene at 99% homology. Primer P1F1 was designed based on the first 21 nucleotides of the ORF of *B. stearothermophilus* P1 lipase gene sequence, whereas primer P1R1 was from the last 21 nucleotides of the same sequence. The combination of these new primers (P1F1 and P1R2) was not able to amplify any PCR product. However, the combination of primer P1R1 with primer Lip F2 was successful and an 800-bp gene fragment was obtained through PCR amplification. The purified PCR product was sent for sequencing and based on the sequence, this lipase gene showed high similarities with most of the *Bacillus* thermostable lipase genes, especially *Geobacillus* sp. T1 (AY 260764) (92%).

Based on the high similarities with *Geobacillus* sp. T1, a new forward primer, F, was designed. The combination of primer F and Lip R2 successfully amplified a PCR product with a size of ∼700 bp. The purified DNA was sent for sequencing and the sequence obtained was later used to design another forward primer which started at the mature lipase gene sequence, which was later used to amplify a complete mature lipase gene (∼1200 bp) when combined with primer P1R1. The 1200-bp PCR product obtained was eluted from the gel and then ligated into a U-overhang vector, pQE-30. Positive transformants showed a cleared zone around the colonies on a tributyrin-LB plate with associated antibiotic agar after overnight incubation at 37 °C. The positive clones were streaked onto triolein-LB agar to screen for true lipase producers and were incubated overnight at 37 °C. The recombinant clones formed an intense blue color on the triolein-LB agar plate (data not shown).

### 2.3. Sequence Analysis of the Thermostable L2 Lipase

**Figure 2.**
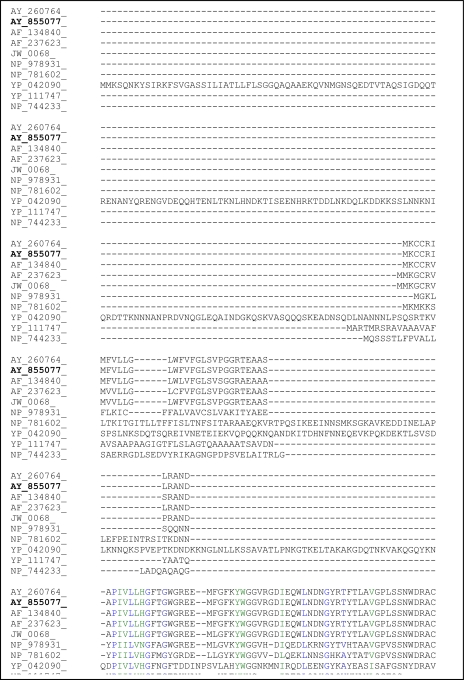

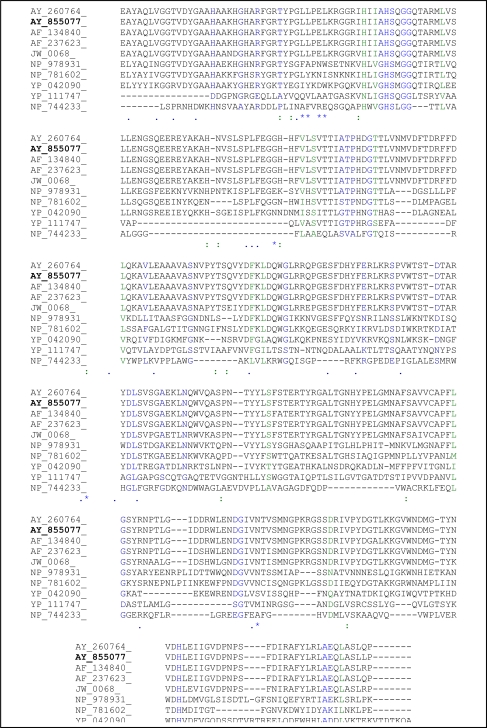
Homology lineup of lipases from several bacteria species. Note: All the lipases were labeled based on their accession numbers submitted to the GenBank. *Geobacillus* sp. T1 lipase (AY 260764); *Bacillus* sp. L2 lipase (AY 855077); *B. thermoleoverans* ID-1 lipase (AF 134840); *B. stearothermophilus* P1 lipase (AF 237623); *B. stearothermophilus* LI lipase (JW 0068); *B. cereus* ATCC 10987 lipase (NP 978931); *Clostridium tetani* lipase (NP 781602); *Staphylococcus aureus* subsp. aureus MRSA252 lipase (YP 042090); *Burkholderia pseudomallei* K96243 lipase (YP 111747) and *Pseudomonas putida* lipase (NP 744233).

The plasmid harboring the L2 gene was sequenced and shown to contain a 1.2 kb sequence coding for lipase. The complete sequence of the ORF of the L2 lipase gene was submitted to GenBank and was assigned accession number AY855077. The amino acid composition within the ORF was determined by the ProtParam Tool of the ExPASy Molecular Biology server [6]. The molecular mass and the isoelectric point (pI) were predicted to be 46.31 kDa and 6.36, respectively. The putative signal peptide cleavage site of *Bacillus* sp. L2 lipase was found to be located between Ala-28 and Ala-29, based on the rules for signal peptide sequences and based on predictions from the SignalP V2.0 Web server [7]. In all lipases, a catalytic triad of Ser, His and Asp (or Glu in a few lipases) is present [8]. Thus, the predicted catalytic triad of *Bacillus* sp. L2 lipase should be formed by His-42, Ser-14 and Asp-345 according to comparisons with the His, Ser and Asp catalytic triad of other *Bacillus* lipases (Figure 2).

### 2.4. Overexpression of the L2 Lipase Gene

**Figure 3.**
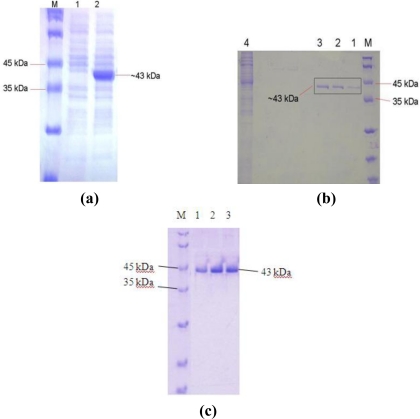
**(a)** SDS-PAGE gel shows IPTG induction of the recombinant lipase; M, Standard protein markers; Lane 1, No added IPTG; Lane 2, 1 mM IPTG; **(b)** SDS-PAGE; M, Standard protein markers; Lane 1–3, Purified fractions; Lane 4, Crude protein; and **(c)** Native-PAGE gels of L2 His-tagged recombinant L2 purified through Ni-Sepharose 6 Fast Flow affinity chromatography; M, Standard protein markers; Lane 1–3, Purified fractions.

High-level expression using the pQE-30 vector is based on the T5 promoter transcription-translation system. The expression of *E. coli* M15[pREP4] harboring recombinant plasmids was rapidly induced by addition of 1.0 mM isopropyl-β-D-thio-galactoside (IPTG) for two hours of induction time (Figure 3a). These expression levels were 172-fold higher than those produced by the native *Bacillus* sp. L2 and the transformed *E. coli* in the absence of IPTG. A higher concentration of IPTG was not used in this experiment because it was assumed that higher concentration of IPTG would result in significant retardation of cell growth, resulting in a lower protein yield [9]. The same IPTG concentration was used by Sinchaikul *et al.* (2001) with a post-induction time of two hours for expression of the thermostable lipase P1 using vector pQE-60 and *E. coli* M15[pREP4] as host cells [10]. In addition, Leow *et al.* (2004) also reported that 1 mM of IPTG was used for expression of thermostable lipase T1 using several vectors, including pET22b(+), pRSET, and pGEX-4T1 [11]. However, Nthangeni *et al.* (2001) reported that only 0.5 mM of IPTG was used to induce the expression of lipase from *B. licheniformis* using pET20b(+) and E. coli JM109 (DE3) as expression vector and host, respectively [12].

### 2.5. Purification of Recombinant L2 Lipase

The recombinant His-tagged L2 lipase was successfully purified to homogeneity by the single-step affinity chromatography method. With this purification strategy, a 76.1% recovery was achieved with a purification of 1.6 fold and a specific activity of 75.8 U/mg. The yield was found to be higher than the yields of either His-tagged *Staphylococcus xylosus* lipase (66% of recovery) or its mutant (71% of recovery) [13]. Although the purification level for THL027 lipase was high (2.6-fold), its recovery was found to be only 27% with a specific activity of 4.5 U/mg, analyzed after using a single step of Sephadex G-100 gel filtration prior to ultrafiltration through a 10 kDa cut-off membrane [14]. The results of SDS-PAGE analysis of the Ni-Sepharose affinity chromatography-purified recombinant L2 lipase are shown in Figure 3b. The oligomeric state of the purified recombinant L2 lipase was predicted to be monomer as L2 lipase was found to be migrated as a single band on both SDS-PAGE and Native-PAGE gels with a relative molecular mass of approximately 43 kDa in Figure 3c.

### 2.6. Effect of pH on Lipase Activity and Stability

**Figure 4.**
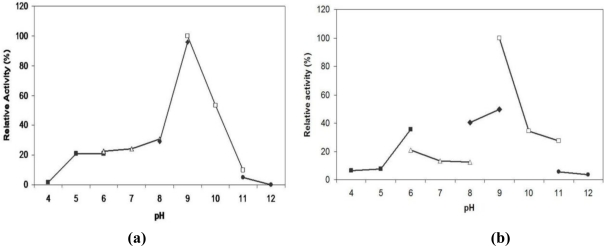
**(a)** pH profile of purified recombinant L2 lipase; **(b)** pH stability of purified recombinant L2 lipase. Symbols used are: (▪) Acetate buffer; (▵) Potassium phosphate buffer; (♦) Tris-HCl buffer; (□) Glycine-NaOH buffer; (•) Na_2_HPO_4_/NaOH buffer.

The effect of pH on enzyme activity was examined in the pH range of 4.0 to 12.0 (Figure 4a). Maximal lipolytic activity towards olive oil was observed at pH 9.0. At pH 10, L2 lipase remained at 50% of its activity and the activity started to decrease tremendously when the lipase was assayed at higher pH levels. L2 lipase also seemed to have low activity at acidic to neutral pH as it only had 20% of its activity at pH 5 to pH 7. The high enzyme activity at pH 9 to 10 may be a result of the Ala for the first Gly-residue in the consensus sequence Gly-X-Ser-X-Gly [15]. The pH-stability of L2 showed that it was fairly stable (>40% of relative activity) at pHs ranging from 8.0 to 10 after being treated for 30 min at 70 °C in both Tris-HCl and Glycine-NaOH buffers (Figure 4b). L2 lipase was most stable at pH 9.0.

### 2.7. Effect of Temperature on Activity and Thermostability Profile

**Figure 5.**
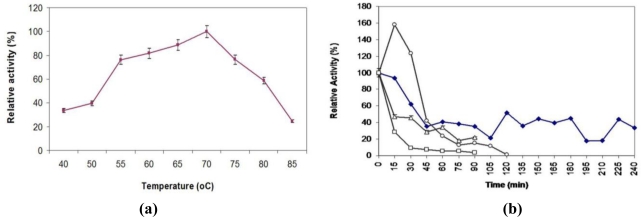
**(a)** Temperature profile of purified recombinant L2 lipase; and **(b)** Thermostability profile of purified recombinant L2 lipase. The L2 lipase was incubated at different temperatures in Glycine-NaOH buffer pH 9 and the residual activity was assayed at 70 °C (optimum temperature). The symbols used are: (□) 80 °C; (▵) 70 °C; (○) 65 °C; and (♦) 60 °C.

L2 lipase was most active in the temperature range of 55 to 75 °C, with more than 75% of its lipolytic activity remaining, and exhibited its maximal activity at 70 °C (Figure 5a). These characteristics indicated that L2 lipase is a thermostable lipase. Studies by Dharmsthiti and Luchai (1999) on THL027 lipase showed that it was highly active at 70 °C even though the lipase molecular mass was relatively higher (69 kDa) than that of the three thermostable lipases mentioned earlier [14]. Lower optimum temperatures were found from other thermostable lipases such as that produced by *Bacillus* strain J33 [16], *Bacillus* sp. RSJ-1 [17] and *Bacillus* A30–1 [18], where their optimum temperatures were around 50 to 60 °C.

The purified L2 lipase was stable at 60 °C with a half-life (t_1/2_) of 2 h (Figure 5b), similar to the characteristics of BTID-B lipase that were reported previously [19]. At the same temperature, L2 lipase retained more than 40% of its original activity for 3 h and 45 min. The thermostability of L2 lipase was higher than that of the previously reported lipase *Bacillus stearothermophilus* L1 [20] which was stable at 60 °C for 30 min. The relatively high stability of this enzyme at high temperatures fits requirements for industrial processes.

### 2.8. Denatured Protein Analysis of L2 Lipase

**Figure 6.**
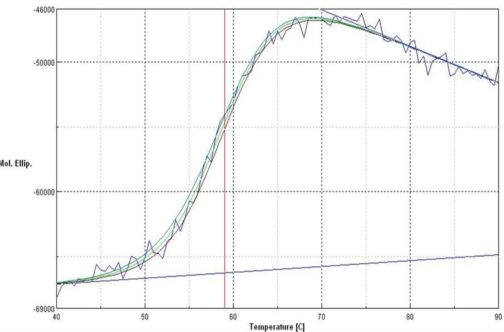
The CD spectra of L2 lipase at 220 nm denatured protein analysis. The fitting lines and thermal bar were indicated in blue and red, respectively.

Figure 6 shows the CD spectra of the L2 lipase measured at various temperatures. From this study, it was found that the melting point, T_m_ for L2 lipase was 59.04 °C. In agreement, the half-life for L2 lipase was around 120 min at 60 °C as the treatment temperature is slightly below its T_m_.

### 2.9. Effect of Metal Ions on Lipase Activity

**Figure 7.**
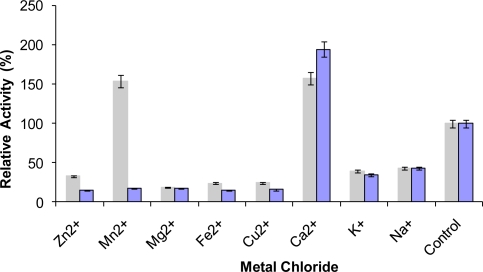
Effect of metal ion on purified recombinant L2 lipase. L2 lipase was preincubated at 65 °C for 30 min with different metal ions at 1 mM ( 
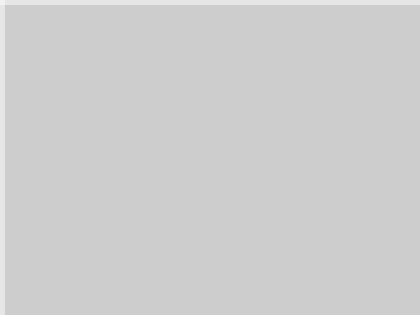
) and 10 mM ( 
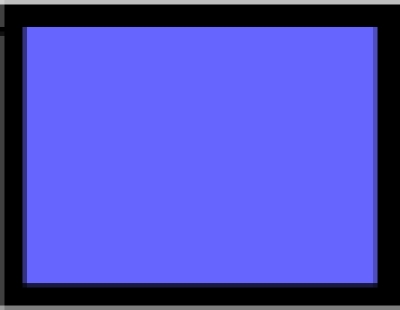
) prior to lipase assay. The remaining activity was determined at 70 °C using olive oil emulsion (1:1, v/v in Glycine-NaOH buffer pH 9) and expressed as a percentage of the activity without the metal chlorides.

Many enzymes contain metal ions to maintain a stable and active structure, where the metal ions are bound strongly to specific binding sites on the surface of the molecules. Stabilization of enzymes by metal ions at high temperatures occurs by metal ion complexing, a process with a favorable entropy factor [21]. The presence of metal ions is believed to have a significant effect on the rigidity and hence the stability of enzymes. In the present study, it was found that the presence of Ca^2+^ ions (1 mM and 10 mM) greatly increased the L2 lipase activity by approximately 100–200% (Figure 7). The L2 lipase activity was also promoted in the presence of 1mM Mn^2+^, K^+^ and Na^+^ by 100–155% of its relative activity. The addition of 10 mM of metal ions such as Zn^2+^, Mn^2+^, Mg^2+^, Fe^2+^ and Cu^2+^ decreased the activity by more than 60%.

### 2.10. Influence of Various Effectors on L2 Lipase Activity

**Figure 8.**
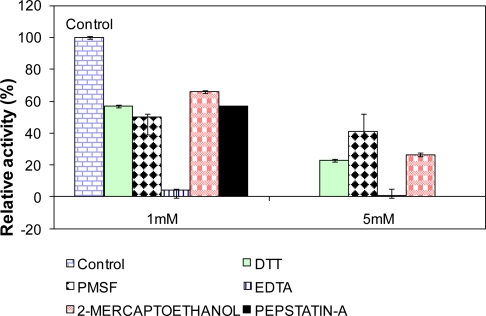
Influence of effectors on purified recombinant L2 lipase activity. The L2 lipase was incubated with 1 mm and 5 mm of effectors (except Pepstatin A) at 65 °C for 30 min prior to lipase assay at standard conditions.

Figure 8 showed the relative activity of L2 lipase after being treated for 30 min at 65 °C with 1% and 5% of various effectors. EDTA treatments (both 1% and 5%) were found to strongly inhibit L2 lipase, especially at 5 mM EDTA, which caused a complete block of activity (0%). This result indicates that the enzyme is a metalloenzyme. Similar inhibition was also reported on the lipases BTID-A and BTID-B [18]. Treatments of L2 lipase with PMSF and pepstatin A also showed strong inhibitory effects (more than 60% inhibition), indicating that the catalytic triad of L2 lipase consists of Ser-His-Asp. DTT and 2-mercaptoethanol showed moderate inhibitory effects to the activity of L2 lipase at a concentration of 1 mM, but significantly high levels of inhibition when a 5 mM concentration was used.

### 2.11. Secondary Structure Prediction of L2 Lipase by Circular Dichroism (CD) Spectral Analysis

**Figure 9.**
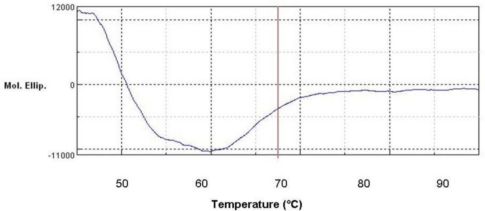
Molecular ellipticity value measurement for L2 mature lipase. Secondary structure prediction of L2 lipase was done by the CD spectra at 220 nm.

One of the most successful applications of CD in characterizing a protein depends upon the remarkable sensitivity of the far-UV to the backbone conformation of proteins to reflect the secondary content of the protein [22]. CD measurements have been widely used to follow the equilibrium between helical structures and unordered conformations [23]. Polypeptide conformations that determine protein secondary structures give rise to circular dichroism spectra [24]. Measuring the CD spectra of L2 lipase allowed rapid determination of the secondary structure content of L2 lipase. The purified L2 lipase was first diluted to a concentration of 0.2mg/ml before subjected to CD and its far UV spectra 190–240 nm were obtained (Figure 9). L2 lipase was determined structurally to be 38.6% α-helix, 2.2% ß-sheet, 23.6% ß-turn and 35.6% random coil.

## 3. Experimental Section

### 3.1. Strains, Plasmids and Growth Conditions

*Bacillus* sp. strain L2 used as the wild-type strain was isolated from a hot spring in Perak, Malaysia [25]. This strain was grown at 70 °C in trypticase soy broth (TSB) and also on a nutrient agar plate for 28 hours. *E. coli* M15[pREP4] (Qiagen, Valencia, CA, USA) was used as a heterologous cloning and expression host for recombinant studies and was grown in LB supplemented with 25 μg/mL (w/v) kanamycin. Plasmid pQE-30 UA (Qiagen, Valencia, CA, USA) was used for cloning and also as an expression vector.

### 3.2. Nucleic Acid Manipulation

Genomic DNA of *Bacillus* sp. strain L2 was extracted using the DNeasy tissue kit (Qiagen, Valencia, CA, USA) according to the manufacturer’s manual. Plasmid DNA was isolated with the Mini Plasmid Kit (Qiagen, Valencia, CA, USA). Extraction of both genomic and plasmid DNA from agarose was performed with the Gel Extraction Kit (Qiagen, Valencia, CA, USA) as specified by manufacturer. Competent *E. coli* M15[pREP4] cells were prepared using the protocol from (Qiagen, Valencia, CA, USA).

### 3.3. 16S rDNA Gene Sequence Amplification

16S rDNA gene sequence was amplified via polymerase Chain Reaction (PCR) using two degenerate universal primers: 16s Forward: 5′-CCG AAT TCG TCG ACA ACA GAG TTT GAT CCT GGC TCA G-3′; and 16s Reverse: 5′-CCC GGG ATC CAA GCT TAC GGC TAC CTT GTT ACG ACT T-3′. The expected PCR product is 1500 bp. Amplification process was carried out in 100 μL of reaction mixture containing 1.5 mM MgCL_2_, 1 × PCR buffer, 0.2 mM dNTP mix, 2 U of Taq DNA Polymerase, 20 pmol of each forward and reverse primers and genomic DNA (50–100 ng). The gene was amplified with a thermocycler (Gene Amp PCR system 2400, Perkin Elmer, Foster, CA, USA). The conditions for the PCR amplification were as follows: pre-denaturation at 94 °C for 4 min, denaturation at 94 °C for 1 min, annealing at 58 °C for 2 min, extension at 72 °C for 1 min, final extension at 72 °C for 7 min and preservation at 4 °C.

The amplified products were detected by electrophoresing 10 μL of PCR product through 1% agarose gel at 70 mA for 30 min and then subjected to staining with ethidium bromide (1 μg/μL) for 15 min and later ligated into TA cloning vector (Invitrogen, Carlsbad, CA, USA,) according to manufacturer’s instructions. After transformation into *E. coli*, the plasmids were extracted and sent for sequencing. A DNA homology search was performed with GenBank database [26].

### 3.4. Phylogenetic Tree Analysis

A phylogenetic tree based on comparison of 16S rDNA sequence of this strain and other strains of *Bacillus* species was constructed. All sequences were aligned with CLUSTAL W 1.75 [27]. Phylogeny tree was constructed in Biology Workbench 2.0 [28]. 16S rDNA sequences of other *Bacilli* were obtained from GenBank database [26].

### 3.5. Isolation of the Lipase Gene

*Bacillus* sp. L2 genomic DNA (diluted 1:10, approximately 10 ng) was used as a template for the PCR amplification of lipase gene fragments, using the degenerate consensus primers listed below with the possible combinations.

Lip F2: 5′-TAG AGA ACG GAA GCC AAG AAG A-3′
Lip R2: 5′-GAG CCG TTC AAA ATA ATG GTC G-3′
P1R1: 5′-TTA AGG CTG CAA GCT CGC CAACTG-3′
F: 5′-CAG AAA ACC CGA CAA TTG CCG-3′
Mature: 5′-GCA TCC CTA CGC GCC CAT GAT-3′

PCR was carried out in 100 μL of mixture containing 1.5 mM MgCl_2_, 1 × PCR buffer, 0.2 mM dNTP mix, 2.5 U Taq DNA Polymerase, 20–30 pmol each reverse and forward primers and DNA template (50–100 ng). The reaction mixture was amplified in a thermocycler (GeneAmp PCR System 2400, Perkin Elmer, CA, USA). The conditions for the PCR amplification were as follows: pre-denaturation at 95 °C for 4 min, denaturation at 95 °C for 1 min, annealing at 55 °C for 2 min for primers Lip F2/Lip R2 and Lip F2/PIRI and 58 °C for 2 min for primers F/Lip R2 and Mature/P1R1, extension at 95 °C for 1 min, final extension at 95 °C for 7 min and preservation at 4 °C.

### 3.6. Cloning of PCR Product

The purified DNA of the amplified mature lipase gene was directly cloned with pQE-30 UA expression vector (Qiagen, Valencia, CA, USA) according to the manufacturer’s instructions. The transformation medium was plated onto separated LB-trybutyrin plates (100 μg/mL ampicillin, 25 μg/mL kanamycin) and incubated at 37 °C overnight. Colonies that produced clearing zones on tributyrin-ampicillin-kanamycin LB agar were isolated and restreaked on triolein-ampicillin-kanamycin agar. Formation of blue zones around the colonies indicated lipase activity.

### 3.7. Sequencing of the Thermostable Lipase Gene

The purified recombinant plasmid was sent for automated sequencing (Institute of Bioscience, UPM). Samples were sequenced using an ABI PRISM 377 Genetic Analyzer (Perkin-Elmer). Analysis of the sequence and database similarity search were done using BLAST from National Center for Biotechnology (NCBI) [29], ExPASy Molecular Biology server [6] and Biology Workbench [28].

### 3.8. Expression of the L2 Lipase Gene

The *E. coli* strain harboring recombinant plasmids was grown for 3 hours in 500 mL bottles containing 100 mL of LB medium supplemented with 100 μg/mL ampicillin and 25 μg/mL kanamycin on a rotary shaker (200 rpm) at 37 °C. The culture was induced with 1 mM of isopropyl-β-d-thiogalactopyranoside (IPTG) at OD_600nm_ ∼ 0.5 for 3 hours. Cultures (10 mL) were harvested by centrifugation and resuspended with 10 mL of 50 mM potassium phosphate buffer (pH 7.0) before sonication (Branson 250 sonifier: output 2, duty cycle 30 and min 2) and cleared by centrifugation (12,000 rpm, 20 min). The clear crude lysate was used in the lipase assay.

### 3.9. Assay of Lipase Activity

The lipase activity was assayed colorimetrically by a method developed by Kwon and Rhee [30]. Culture filtrate (1 mL) was shaken with 2.5 mL of olive oil (70% oleate residues) emulsion (1:1 v/v) and 20 μL of 0.02 M CaCl_2_ in a water bath shaker at an agitation rate of 200 rpm. The emulsion was prepared by mixing together an equal volume of olive oil (Bertoli, Italy) and 50 mM phosphate buffer with a magnetic stirrer for 10 minutes. The reaction mixture was shaken for 30 min at 70 °C. The enzyme reaction in the emulsion system was stopped by adding 6N HCL (1 mL) and isooctane (5 mL), followed by mixing using a vortex mixer for 30 s. The upper isooctane layer (4 mL) containing the fatty acid was transferred to a test tube for analysis. Copper reagent (1 mL) was added and again mixed with a vortex mixer for 30 s. The absorbance of the upper layer was read at 716 nm. Lipase activity was measured by measuring the amount of free fatty acid released from the standard curves of free fatty acids. One unit of lipase activity was defined as the amount of enzyme releasing 1 μmole of fatty acid per minute.

### 3.10. Effect of Different Concentrations of Inducer (IPTG) on Crude Lipase Expression

The effects of using different concentrations of inducer (IPTG) was assessed by inducing the *E.coli* M15[pREP4] cells that had recombinant plasmids with different concentrations of IPTG: 0 mM, 0.25 mM, 0.5 mM, 0.75 mM and 1.0 mM at OD_600nm_ ∼ 0.5 for 3 h of induction time at 37 °C. The best IPTG concentration was used for further studies.

### 3.11. Time-Course Analysis of Crude Lipase Expression

Time-course studies were performed by collecting the cell pellet at different post-induction times: 0, 3, 6, 9, 12, 18 and 36 h at 37 °C. The studies were also repeated with a shorter time frame (0 to 6 h). In this study, 1 mM of IPTG was used as an inducer. The best post-induction time was chosen for further studies.

### 3.12. Purification of Recombinant Lipase L2

The cell pellet from a 200 mL culture was suspended in 20 mL of binding buffer (20mM sodium phosphate, 500 mM NaCl, 20 mM imidazole) before sonication (Branson 250 sonifier: output 2, duty cycle 30 and min 2) and cleared by centrifugation (10,000 rpm, 30 min). The clear lysate was collected and used as the crude enzyme in purification using a Ni-Sepharose affinity chromatography column. The crude L2 lipase solution was purified to homogeneity by a one-step purification protocol using immobilized metal affinity chromatography (IMAC). The purification procedure was performed using an AKTA purifier system (Amersham Bioscience, USA) according to the manufacturer’s instructions. Ni-Sepharose 6 Fast Flow (5 mL) was used as the matrix in an XK 16/20 (GE Healthcare, Salt Lake City, UT, USA) column. Equilibration was performed using binding buffer (20 mM sodium phosphate, 500 mM NaCl, 20 mM imidazole).

An aliquot of 20 mL of filtered crude recombinant L2 lipase was loaded in the equilibrated column at a flow rate of 0.5 mL/min. The column was washed with five column volumes of binding buffer. The recombinant L2 lipase was eluted with 10 CV of elution buffer (20 mM sodium phosphate, 500 mM NaCl, 500 mM imidazole, pH 7.4) using a linear gradient of imidazole ranging from 20 to 500 mM. The eluted fractions were subjected to SDS-PAGE analysis, lipase assay and Bradford assay.

### 3.13. SDS-PAGE Analysis of Bacterial Protein

SDS-PAGE was carried out on 12% running gel by using the method of Laemmli (1970) [31]. A broad range of prestained protein standard (MBI Fermentas; St. Leon-Rot, Germany) was used as a molecular mass marker. After electrophoresis, gels were stained with Coomasie Brilliant Blue R-250 (BioRad, Hercules, CA, USA).

### 3.14. Effect of pH on Lipase Activity and Stability

Enzymatic activity was measured at various pH values (pH 4–12) for 30 min at 70 °C. The substrate (olive oil) was prepared in 50 mM of various buffers; acetate buffer (pH 4.0–6.0), potassium phosphate buffer (pH 6.0–8.0), Tris-HCl buffer (pH 8.0–9.0), glycine-NaOH buffer (pH 9.0–11.0), and Na_2_HPO_3_/NaOH buffer (pH 11.0–12.0). The effect of pH on lipase stability was determined by incubating aliquots of L2 lipase in buffers of different pH values for 30 min at 70 °C. Residual activity was assayed using the Kwon and Rhee method.

### 3.15. Effect of Temperature on Lipase Activity and Stability

The optimum temperature for lipase activity was determined over the range of 40–85 °C. The substrate was equilibrated at the required temperature for 5 min before the addition of enzyme. The effect of temperature on lipase stability was determined by incubating aliquots of the purified L2 lipase in Glycine-NaOH pH 9.0 at 60 °C to 80 °C in intervals of 5 °C. The residual activity was measured at the optimum temperature (70 °C).

### 3.16. Denatured Protein Analysis

The variable temperature measurement of L2 lipase was performed by employing 10 mm cell after checking the circular dichroism (CD) value at 220 nm. The warm-up period was 50 to 95 °C, and the step was 1 degree per minute. The wavelength was set to 220 nm. The concentration was 1 mg/mL and top of the cell was completely closed using a cap. Data pitch, bandwidth, response, scanning speed, and accumulation were set to be 0.1 degree, 1 nm, 8 seconds, 1 degree per minute, 8 times, respectively.

### 3.17. Effect of Metal Ions on Lipase Activity

The activity of the purified L2 lipase was studied following incubation with 1 mM and 10 mM concentrations of various metal chlorides (Na^+^, K^+^, Mg^2+^, Ca^2+^, Fe^2+^, Mn^2+^, Zn^2+^, and Cu^2+^) in 50 mM Glycine-NaOH buffer pH 9.0 at 65 °C for 30 min. The residual activity was determined at 70 °C using olive oil emulsion and expressed as a percentage of activity without the metal chlorides.

### 3.18. Influence of Various Effectors on Lipase Activity

The influence of various effectors on purified L2 lipase activity was determined by incubating the purified L2 lipase at 65 °C for 30 min in 50 mM Glycine-NaOH buffer pH 9.0 with dithiothreitol (DTT), 2-mercaptoethanol, phenylmethylsulfonyl fluoride (PMSF), ethylene diaminetetra acetic acid (EDTA), and pepstatin A at different concentrations (1 mM and 5 mM) except for pepstatin (1 mM). The residual activity was measured at 70 °C and expressed as percentage of activity without inhibitors.

### 3.19. Circular Dichroism (CD) Spectral Analysis of L2 Lipase

Purified L2 lipase in sodium phosphate buffer (pH 8.0) was analyzed with spectropolarimeter J-810 (Jasco, Tokyo, Japan) for CD spectral analysis. The warm-up periods of 50 to 95 °C and wavelength scan of 180 to 250 nm were taken into consideration.

## 4. Conclusions

In this study, we reported the identification of a newly isolated thermostable lipase producer, *Bacillus* sp. L2, the PCR cloning, sequencing and over-expression of a functional form of thermostable lipase L2, as well as purification and characterization studies. The full sequence of the L2 lipase gene was analyzed. A high-level expression was also successfully achieved by using the pQE-30/E. coli M15[pREP4] expression system. This system worked efficiently and overexpression of recombinant L2 was successfully carried out with a 178-fold higher activity compared to crude native L2 lipase. So far, no other lipases have been reported to successfully use this expression system. Therefore, we conclude that L2 lipase is the first lipase for which this system has been successfully used.

The recombinant L2 lipase was easily recovered via a single-step affinity chromatography purification method with high purity and high yield purification, leading to a low-cost method for preparation of this enzyme. The characterization of this enzyme resulted in some interesting findings, such as its thermostability profile and its wide range of pH stability, indicating that L2 lipase has significant potential for commercialization as a biocatalyst for industrial purposes, specifically, in detergent industries and food industries that use esterification process at high temperature. Therefore, in comparison with other commercially available enzymes, the use of L2 lipase for industrial applications seems promising as it is more stable at higher temperatures.
